# A methodological framework to operationalize climate risk management: managing sovereign climate-related extreme event risk in Austria

**DOI:** 10.1007/s11027-016-9713-0

**Published:** 2016-04-19

**Authors:** Thomas Schinko, Reinhard Mechler, Stefan Hochrainer-Stigler

**Affiliations:** 1 International Institute for Applied Systems Analysis (IIASA), Schlossplatz 1, 2361 Laxenburg, Austria; 2 Wegener Center for Climate and Global Change, University of Graz, Graz, Austria; 3 Vienna University of Economics and Business, Wien, Austria

**Keywords:** Climate adaptation, Disaster risk reduction, Climate risk management, Iteration, Uncertainty, Extreme events, Flood risk, Risk layering

## Abstract

Despite considerable uncertainties regarding the exact contribution of anthropogenic climate change to disaster risk, rising losses from extreme events have highlighted the need to comprehensively address climate-related risk. This requires linking climate adaptation to disaster risk management (DRM), leading to what has been broadly referred to as climate risk management (CRM). While this concept has received attention in debate, important gaps remain in terms of operationalizing it with applicable methods and tools for specific risks and decision-contexts. By developing and applying a methodological approach to CRM in the decision context of sovereign risk (flooding) in Austria we test the usefulness of CRM, and based on these insights, inform applications in other decision contexts. Our methodological approach builds on multiple lines of evidence and methods. These comprise of a broad stakeholder engagement process, empirical analysis of public budgets, and risk-focused economic modelling. We find that a CRM framework is able to inform instrumental as well as reflexive and participatory debate in practice. Due to the complex interaction of social–ecological systems with climate risks, and taking into account the likelihood of future contingent climate-related fiscal liabilities increasing substantially as a result of socioeconomic developments and climate change, we identify the need for advanced learning processes and iterative updates of CRM management plans. We suggest that strategies comprising a portfolio of policy measures to reduce and manage climate-related risks are particularly effective if they tailor individual instruments to the specific requirements of different risk layers.

## Introduction

Much of the concern about the impacts of climate change is related to projected shifts in intensity, duration, and frequency of climate-related extremes such as floods and droughts (EEA 2014). Science has shown climate change to alter the frequency, duration, and intensity of many natural hazards globally—heatwaves, droughts, and heavy precipitation (IPCC 2014)—as well as to modify heavy precipitation events on local to regional scales (APCC 2014). There is a long history of managing climate-related and geophysical-driven extremes via disaster risk management (DRM).^1^ Recently, the Intergovernmental Panel on Climate Change’s (IPCC) Special Report on Managing the Risks of Extreme Events and Disasters to Advance Climate Change Adaptation (SREX) (IPCC 2012) and 5th Assessment Report (IPCC 2014), as well as other policy-relevant publications (e.g., UNISDR 2015), identified important synergies between DRM and climate change adaptation (CCA) with regard to risk drivers, policy instruments and actors, and made a call for further linking agendas for development practice and planning at the sub-national, national, and international levels (IPCC 2012; 2014; Saito 2013; UNISDR 2015).

International disaster risk and climate policy has followed suite and the Third United Nations (UN) World Conference on Disaster Risk Reduction in Sendai in early 2015, with climate change being among its four priorities for action on local, national, regional, and global levels, emphasized synergies between understanding risk, strengthening risk governance, investing in resilience, enhancing preparedness (UN 2015). Recently, the climate negotiations at the United Nations Framework Convention on Climate Change’s 21st Conference of the Parties (COP21), which resulted in the Paris Agreement, stressed the need for fostering national adaptation planning, including a broad assessment of climate change impacts and vulnerability; this would include comprehensive risk assessment and management in order to deal with climate-related risks (UNFCCC 2015).

In line with the attention being paid to climate risk, there has been discussion concerning appropriate methodological approaches for assessing and managing climate risks. In the IPCC’s 5th Assessment Report (IPCC 2014) Chambera et al. (2014) see increasing need to consider risk in adaptation policy and evaluation, and Jones et al. (2014) suggested climate risk management (CRM) as a broad formula for research, policy, and practice to concurrently tackle climate adaptation and disaster risk in order to reduce negative impacts on the natural environment, human society, and economies. Watkiss et al. (2014) identified climate risk management as a blueprint for early action on climate-related extremes—addressing the current adaptation deficit in the short term, and mainstreaming climate change into medium-term climate adaptation. Mechler et al. (2014) suggested that CRM means comprehensively reducing, preparing for, and financing climate-related risk, while tackling the underlying risk drivers, including climate-related and socio-economic factors. They advise distinguishing between frequent (average) and infrequent (fat-tailed) risks, leading to the notion of risk layering, whereby policies tackling climate-related risk are aligned with the return period of impacts. Patt (2013) also identifies the significant benefits of CRM for longer-term climate adaptation by building up experience through the linking of climate-relevant science to decision-making on shorter-term risks.

Taking into account the multiplicity of risks to manage, the transdisciplinary nature of the problem at hand (see e.g., Mustelin et al. 2013; Bréthaut and Hill Clarvis 2015), the complexity of social–ecological systems (Lavell et al. 2012), as well as the different framing and decision-making contexts (Berkhout et al. 2014) and information needs of stakeholders (Hanger et al. 2013), numerous methods may need to be incorporated into a CRM process. Organizing processes such as these implies tackling fundamental epistemological questions involving uncertainty, as well as learning theories for reducing these uncertainties. In the context of climate change, Jones et al. (2014) suggest distinguishing between CRM assessment methodologies and responses based on the notions of complexity and uncertainty. In a similar vein, Lavell et al. (2012) propose different ways of learning in order to generate different levels of knowing. Accordingly, for simple risks—characterized by relatively low uncertainty in terms of occurrence and outcomes, as well as linear cause-effect relationships—, standard analytical, expert-centric techniques (such as risk modelling) would be suitable for deriving (and over time, providing there was an increase in knowledge, improving) estimates of future risks, which would in turn be communicated to key stakeholders. Complicated risks, defined by uncertainty in outcomes and frequency would need to see strong collaborative and iterative stakeholder interaction, including negotiation of mental and analytical models. Finally, complex risks, characterized by deep uncertainty and contested outcomes, require strong deliberative and adaptive exercises to foster shared understanding and ownership.

Although there are some early policy examples of considering managing risk as an element of national-level adaptation policy and implementation, these have often either dealt with sectors such as water, agriculture, and health, rather than identifying the synergies with disaster risk management (EEA 2014), been associated with institutional barriers (separate and non-cooperation agencies for DRM and CCA) (Mimura et al. 2014), or informational challenges for properly representing variability using probabilistic metrics (Watkiss et al. 2014). Overall, as the CRM formula suggested has been rather broad, clarification is needed as to which risks and decision-contexts CRM can be applied to, as well as to what methodological innovation this entails.

The objective of this study is to identify key lessons in the literature on CRM and to then apply them in an empirical context to evaluate their usefulness for informing policy and practice. To that end, we develop a methodological approach to CRM in the decision context of sovereign (public sector) climate-related risk. We then apply and test this approach in Austria, a country that has been subject to recurrent large-scale flooding, creating substantial fiscal stress. Based on the results of this Austrian case study, and in collaboration with Austrian stakeholders, we co-generate a generic iterative framework that can be used for applying CRM in a wide range of decision contexts.

We find that a CRM framework building on multiple lines of evidence and a variety of methods can, generally speaking, be useful for informing risk management decisions in the short-medium term by addressing the existing adaptation deficit. To deal with the substantial uncertainties associated with risks from future climate change, and with future contingent climate-related fiscal liabilities expected to increase substantially due to socioeconomic developments and climate change, we identify the need for advanced learning and an iterative approach to CRM. We distill major building blocks of a generic iterative CRM strategy applicable to other contexts with similar risks by building on the evidence generated in this case study. In particular, we suggest that strategies comprising a portfolio of policy measures to reduce and manage climate-related risks at the national and international level are particularly effective if they tailor individual instruments to the particular requirements of different risk layers.

## Building blocks of sovereign CRM

Progress in various policy-focused and methodological discourses needs to be taken into account, when considering operationalizing CRM.

### Shift towards pro-active policy to manage disaster risks

Over the last few decades, there has been a paradigm shift in the choice of policy instruments to address disasters, thus moving towards a more pro-active approach, and putting a stronger emphasize on ex ante DRM (Linnerooth-Bayer et al. 2005). Understanding that disasters are not acts of God, but largely unnatural has drawn attention to the need for fostering pro-active risk management in lieu of reactive response (relief and reconstruction) (World Bank and United Nations 2010). Risk management systems, which strive to reduce, pool, and financially share risks, have been devised and in many instances employed. This new notion of pro-active DRM relates to the concept of pro-active—or planned—adaptation in the climate change discourse. In contrast to re-active climate adaptation, which can be described as the gradual coping with consequences over time, pro-active climate adaptation refers to actions which allow to prepare for risks before events materialize. This kind of anticipatory approach is especially important for adapting to the impacts of extreme weather events, such as floods and droughts.

### Dealing with government risk in the balance sheets

Although comprehensive disaster risk management requires joint efforts by the private and public sector, to date it has been the public sector risk that has played a more significant role in the application of proactive risk management approaches. Governments’ central position in DRM is due to the fundamental role it plays in providing goods and services and redistributing income, particularly in times of need (Mechler 2004). For example, governments own and allocate public goods (e.g., infrastructure) which are exposed to disaster risk, but they also absorb private sector risk if private coping mechanisms (e.g., disaster insurance) are non-existent, incomplete or break down, and in the case of partial market failure, may also step in to provide those coping services; furthermore, governments provide support to those who are unable to protect themselves. Consequently, when unprecedented hazard events occur the burden falls mainly on governments.

While losses for extreme hazard phenomena can be high, for governments disaster risk usually constitutes a contingent liability, i.e., costs that accrue only in the case of an event. As a result, governments have often ignored catastrophic risks in their planning, and implicitly or explicitly exhibit risk-neutrality (Mechler 2004; Gurenko 2004). This can be justified if risk neutrality prevails (i.e., risks can be absorbed and refinanced relatively easily (Arrow and Lind 1970). Nevertheless, in line with the global shift to a more pro-active approach, many developing countries have become more aware of their risk-averse position and started to plan and budget accordingly. Faced with increasing risk in the short-medium term and climate change in the medium-long-term, and aware that contingent liabilities may cause considerable fiscal stress, there is an indication that OECD countries are also beginning to take action (e.g., USA, Austria) (Mechler and Hochrainer-Stigler 2014).

Progress in public sector risk-planning has been achieved by using the tools available to systematically assess and manage risks in the fiscal balance sheet (Polackova Brixi and Schick 2002; Mechler and Hochrainer-Stigler 2014). Many developed countries, including Austria (our main point of reference in this case) already have an instrument, such as a disaster fund, in place to take some of the implicit climate risks out of their balance sheets and make these contingent climate-related liabilities more explicit.

### Climate change: from burning embers towards calculated risk

Conceptualizations and applications of risk in the context of climate impacts and adaptation research are not new and have been part and parcel of discussions on dealing with uncertainty (e.g., Swart et al. 2009). However, debate in this case was largely confined to the epistemological, as opposed to the instrumental debate seen in the DRM domain (IRGC (International Risk Governance Council) 2005). In order to inform Article 2 of the United Nations Framework Convention on Climate Change (UNFCCC) on the question of dangerous interference with the climate system (UN 1992), the IPCC developed the aggregate analysis and visualization of the so-called five reasons for concern, known colloquially as burning embers, and this became an iconic representation of key risks (Smith et al. 2001, 2009). Most significantly this visualization informed both the 2-degree global warming target adopted at COP 16 to the UNFCC and later the 1.5 degree target adopted by the Paris agreement. Over the last few years, the role of risk in responses to climate change has attracted more attention, particularly with the publication of IPCCs 5th Assessment Report. Although it generated intense discussion among working groups I, II, and III, it was working group II that particularly fostered thinking on climate risk and risk management. The foundational chapter by Jones et al. (2014) suggested a balanced perspective organized around three framings of risk, each with their own particular emphases: (1) Idealized risk—the conceptual framing of climate change risk under the UNFCCC as dangerous anthropogenic interference with the climate system, represented by the reasons for concern as the dominant framework for informing mitigation and the 2-degree target; (2) perceived risk—the subjective judgment people make about an idealized risk for informing adaptation; and (3) calculated risk—the product of a quantitative risk analytical exercise based on a mixture of historical (observed) and theoretical information for informing both adaptation and mitigation questions. Jones et al. (2014) suggest that while the focus has mainly been confined to (1), (2), and (3) are also beginning to generate more attention.

### Managing risk and fostering learning for dealing with uncertainty

In addition to linking DRM and CCA in practice in order to facilitate comprehensive CRM, more recently, the literature has moved towards a more reflexive-participative approach. Acknowledging the uncertainties and complexities inherent in social–ecological systems interacting with climate-related risks, analysts have started to emphasize iterative and adaptive learning (see, e.g., O’Brien et al. 2012; Mochizuki et al. 2015). Lavell et al (2012) suggest a learning loop framework (see also Kolb and Fry 1975; Argyris and Schön 1978; Keen et al. 2005) that integrates different learning theories, such as experiential learning (Kolb 1984), adaptive management (Holling 1978) and transformative learning (Mezirow 1995). This framework distinguishes three different loops according to the degree these processes support transformational change of CRM strategies.

**Fig. 1:**
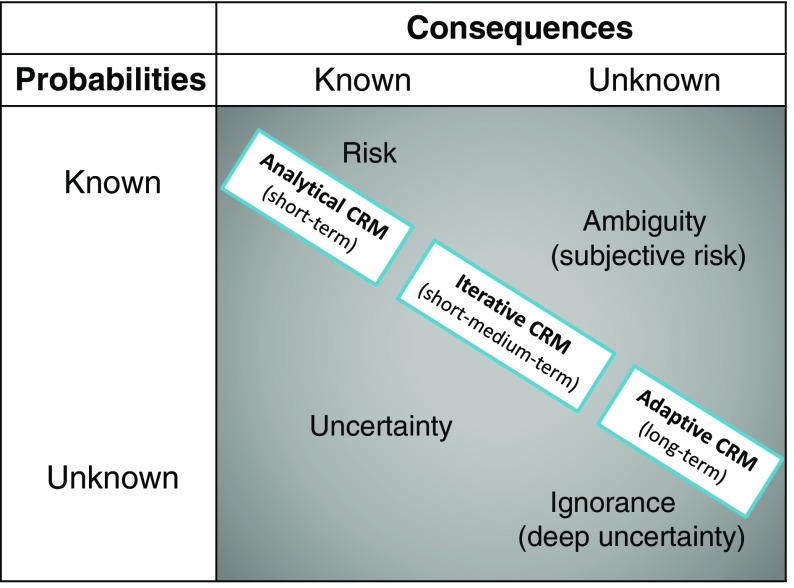
Three CRM approaches—analytical CRM, iterative CRM, and adaptive CRM—for dealing with climate-related risk

Figure 1 suggests three different CRM approaches for dealing with climate-related risks under uncertainty that are applicable in the short, medium, and long term, respectively. The table is organized around the Knightian distinction (1921) of risk, uncertainty, ambiguity, and ignorance, defined as the knowledge of probabilities and outcomes. DRM, with a long and proven track record for tackling disaster risks, and characterized by modeled probabilities and consequences, is often considered the appropriate short-term entry point. While shorter-term (less than a decade) risk assessment and management is characterized by difficulties in estimating the magnitude and likelihood for extreme events and their impacts—particularly true when focusing on the influence of climate change—probabilities and consequences are known with a certain degree of uncertainty (Lavell et al. 2012). Elaborate techniques are necessary for short-term estimation; yet, may be considered relatively simple (in terms of learning), as defined by Jones et al. (2014). In the medium-term (one to two decades into the future), uncertainty, both in terms of probability and outcomes, becomes more prevalent. Thus, a strong and conclusive case for climate change increasing extreme event risks beyond the physical impacts still needs to be made (Bouwer 2011; IPCC 2012; Mechler et al. 2014). So far, the increase in losses has been primarily attributed to socioeconomic trends and rising exposure of people and capital at risk, while at the same time acknowledging that an influence of climate change on trends in losses cannot be excluded (IPCC 2012). Climate risk may therefore be considered complicated, and requires a stronger collaborative and iterative stakeholder interaction, including reframing both learning and management processes, as well as mental and analytical models (Lavell et al. 2012). Finally, in the long-term (i.e., beyond 2050), risks become deeply uncertain and complex in terms of contested outcomes, which requires strong deliberative and adaptive exercises to foster transformative learning towards a shared understanding and ownership (Lavell et al. 2012).

### Policy and decision context for CRM in Austria

Austria provides an opportunity to develop and test these CRM propositions. Exposed to various hazards, such as flooding, drought, and avalanches, Austria suffered losses of several billion Euro in riverine floodings in 2002, 2005, and 2013, leading to substantial stress on private and public financial reserves. At the same time, concern about climate change ranks high among Austrian stakeholders, and efforts involving the research, policy, and practice communities have been undertaken to understand the scope of future climate risk, as well as proper risk management and adaptation responses. Whether and how to link DRM and CCA, however, is yet to be explored.

As one of the first bodies to conduct a comprehensive national assessment of climate change, the Austrian Panel on Climate Change (APCC) showed in 2014 that warming in Austria has been more severe than the global average, that risks are bound to increase and that overall there is a need to upgrade adaptation efforts (APCC 2014). More recently, in 2015 a country-wide assessment of the costs of climate change was published, detailing the significant financial implications of unmitigated climate change for public and private actors, amounting to about a billion Euro already today (Steininger et al. 2015). In terms of policy engagement, in 2012, Austria developed its national adaptation strategy (BMLFUW 2012), which co-generated options with a large set of stakeholders, and identified several possible alternatives for action. Protection from natural hazards and disaster risk management are two of 14 different activity categories which are detailed in the climate adaptation strategy and which we will examine here in the context of a more comprehensive discussion of CRM.

## Towards a methodological approach for operationalizing CRM for policy and practice

Due to the transdisciplinary nature of the problem at hand, the inherent complex dynamics of social–ecological systems, as well as different frames and decision-making contexts of stakeholders, the operationalization of CRM, based on the essential building blocks outlined above, requires a methodological approach comprising of various research methods. Hence, we suggest a methodological approach for operationalizing this CRM concept to consist of, at least, the following elements: assessment of literature on current CCA practices dealing with extreme events and natural hazards; assessment of expert and stakeholder views and knowledge via interviews and workshops; empirical assessment of public budgets; and forward-looking, analytical assessment employing climate-risk-based economic modeling.

### Review of disaster statistics and stakeholder consultation

A comprehensive literature review as well as a broad stakeholder dialogue is crucial to monitor recent developments in climate science, to understand and monitor the current framing of a country’s CRM practice (e.g., what adaptation instruments are already in place) and to anticipate potential future developments given the specific current political and institutional framework conditions. Disaster statistics are of immediate importance for monitoring current levels of climate risks. A systematic collection of information on risk management expenditure, in combination with data on disaster losses, allows policy makers to evaluate the effectiveness of implemented measures in reducing the negative impacts of disaster events and eventually to assess the level of resilience against current and future disaster risks. Moreover, disaster statistics increase transparency and may contribute to the promotion of disaster risk management within a country. However, most countries do not have a central repository (such as national accounts) that clearly distinguishes and accounts for disaster risk reduction (DRR) expenditures. Moreover, if data does exist, no clear cut distinction between ex ante and ex post DRR measures can be drawn and to an even lesser degree expenditures relevant for CCA can be identified. Hence, very often the only way to gather empirical expenditure data regarding current and past CRM expenditures is to directly address those federal agencies currently in charge of implementing and financing DRR measures.

### Modelling disaster risk and fiscal stress testing

As a cornerstone of our methodological approach to CRM we employ the CATastrophe SIMulation (CATSIM) framework. CATSIM was designed to evaluate public sector financial disaster risk management strategies and illustrate the trade-offs and choices a country has to make in managing the risks of catastrophic disasters (see Hochrainer-Stigler et al. 2013). CATSIM uses probabilistic modelling of disaster risk to understand the current and future stress imposed on the fiscal position. Risk has to be evaluated at national levels. This task is complex and usually data as well as resource intensive. In Austria, for example, several flood hazard models on local scales exist, however, currently only two flood risk modelling approaches provide country-level flood loss distributions (for a more detailed discussion on flood hazard modelling see Appendix).

CATSIM follows the common practice in catastrophe models and evaluates monetary catastrophe loss as a function of hazard, exposure, and vulnerability modules (Grossi and Kunreuther 2005). Losses are summarized with the help of risk metrics or loss distributions, which inform about the probability that losses do not exceed a given level. In more detail, we perform a copula-based approach to derive probabilistic flood risk estimates on the country level for Austria. We apply a structured coupling of probability loss distributions on the basin scale (derived from the GIS-based distributed model for river basin scale water balance and flood simulation (LISFLOOD); see van der Knijff et al. 2010; Rojas et al. 2012). Dependencies between river basins in Austria are estimated using different copulas (e.g., Clayton, Frank or Gumbel) and are based on maximum river discharges for the period 1990–2011. Afterwards, the loss distributions from each basin are coupled using the given copulas and a minimax ordering approach to finally derive a loss distribution at the country level. The details of the copula methodology, which has been recognized as most appropriate to avoid underestimation of extreme risk (Jongman et al. 2014), and a general algorithm to perform such coupling can be found in Timonina et al. (2015).

**Fig. 2:**
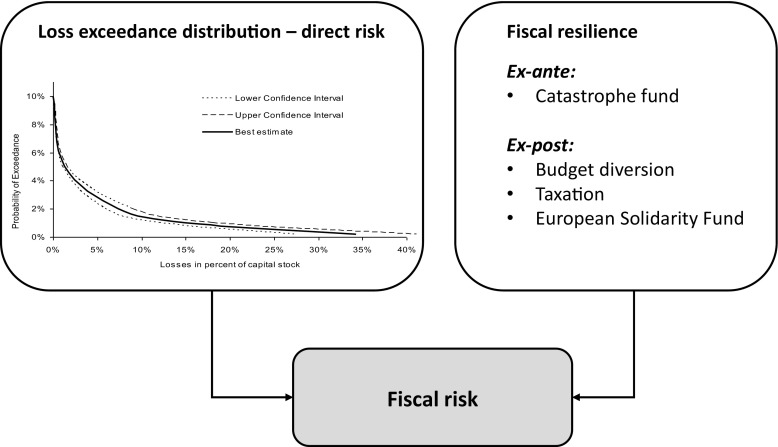
Modeling fiscal risk as a function of losses (direct risk) and fiscal resilience by employing the CATSIM framework. Source: adapted from Hochrainer-Stigler et al. 2013

As a further key step in the CATSIM framework, losses are conjoined with an estimate of fiscal resilience (broadly speaking, budget flexibility) to identify fiscal stress (termed fiscal risk), which provides the basis for evaluating fiscal instruments for managing this risk (Fig. 2).

### Dynamically addressing different layers of risk

In a next step, modelling results can be evaluated in order to reflect on any dynamic change to the loss distribution (the new normal)—characterized by emerging natural hazard and socioeconomic thresholds by employing a risk layering lens. As there are different kinds of climate-related risks, frequent events with only minor impacts vs. infrequent, but destructive events, it is advisable to employ a varied portfolio of instruments, each carefully chosen to be applicable for a certain layer of climate-related risk (Mechler et al. 2014). By contrasting the potential future economic losses due to climate risks against the public resources available for absorbing these risks to assets, the relevant layer of risk at which a specific country might experience fiscal stress in the future, and concrete options to remedy this situation, can be identified.

## Results: evidence and insights from testing the methodology for the case of Austria

We present results obtained by testing the CRM approach, methods, and tools within a broad stakeholder process in Austria. By linking up with key stakeholders in order to conduct budget analyses and expert interviews as well as an expert workshop, we were able to estimate current and future flood risk (as well as any changes therein) according to different layers of risk as well as fiscal consequences of contingent disaster risk liabilities in Austria. Overall, this allowed for shedding light on key economic aspects of the Austrian disaster risk management and climate change adaptation policy and practice.

Employing this comprehensive methodological approach to concrete case studies contributes to identifying major building blocks for the practical implementation of a CRM strategy or the iterative update of a CRM strategy already in place. These insights are not only relevant for the specific country under consideration but may also inform the development of a generic iterative CRM strategy applicable to different national and international decision contexts, which we discuss below.

### Collaborating with stakeholders: DRM as early adaptation in Austria

Semi-structured open-ended interviews with relevant Austrian stakeholders provided insights regarding the framing of the current DRM practice in Austria and helped to identify current and past public expenditures for DRM. A first difficulty was to identify the relevant administrative bodies, as there is no uniform regulation in the Austrian legislation concerning the protection from natural hazards, resulting in a fragmentation of responsibilities. Based on a comprehensive literature review (see, e.g., Rechnungshof 2008) we identified the key institutions dealing with catastrophic impacts and risks of natural hazards in Austria as being located in three ministries—the Federal Ministry of the Interior, the Federal Ministry of Finance and the Federal Ministry of Agriculture, Forestry, Environment and Water Management (BMLFUW). Within these ministries, we identified those public agencies which are responsible for the implementation and the funding of DRR measures in Austria as our prime target group for the expert interviews.

The expert interviews focused on questions regarding both, the status quo and the medium term future of the Austrian DRM practice. As a follow-up to the expert interviews, a stakeholder workshop brought together researchers and relevant experts from the different public agencies involved in the Austrian DRM practice. The aim of this workshop was to discuss the empirical findings regarding the public costs of DRR and CCA, the potential future impacts of climate-related disasters on the fiscal position in Austria and to identify potential entry points for an iterative and robust CRM strategy in Austria. The expert interviews and workshop with stakeholders from the key institutions in the Austrian DRM practice clearly showed that in the current Austrian DRM practice climate change considerations do not play a major role and are not explicitly taken into account in the deliberations by the public agencies responsible for the implementation of DRR measures in practice. As the main reason for that the interview partners stated the lack of a scientifically proven link between the climate change signal and extreme weather events, such as floods, for Austria. In contrast to some German regions (Baden-Württemberg and Bavaria), where hydrological studies (focusing on large-scale weather patterns) have shown a link between climate change and an increase in the frequency and intensity of extreme flood events (Hennegriff et al. 2006; Hennegriff and Kolokotronis 2007), no statistically significant link has been identified for Austria until today (Schöner et al. 2010; Prettenthaler et al. 2015). On the other hand, there is high confidence that today’s and future losses are rising as more assets and people are moving in harm’s way also in Austria (Prettenthaler et al. 2015). Hence, the interviewed experts argued, no explicit implications for the current DRM practice, such as designing and dimensioning of preventive measures, can be derived from existing climate model results, while socioeconomic developments leading to higher exposure require careful attention. In addition to the fact that climate change does not explicitly influence decisions on or conceptions of risk management measures in Austria, there is also no clear consensus in the Austrian DRM practice which public expenditures can be regarded as relevant for CCA.

Due to these reasons, no information on explicit public expenditure for CCA is currently collected and provided in the areas of DRM and the protection from natural hazards. However, the interview partners’ statements on the topic made clear that the Austrian DRM community does not neglect the potential impacts of climate change on future natural hazards. Even though they are not explicitly taken care of at the moment, climate change considerations are implicitly incorporated in the Austrian DRM practice. For example the implementation of no-regret and low-regret options can, according to the IPCC’s SREX report, be seen as starting points for adaptation, as they have the potential to offer benefits now and lay the foundation for addressing projected changes in exposure, vulnerability, and climate extremes. (IPCC 2012; Watkiss et al. 2014) Moreover, by continuously reviewing and integrating new scientific knowledge on climate change (e.g., emerging early trends and changes in variability that exacerbate existing risks or create new risks) the practitioners are adjusting their decisions over time with evidence.

Hence, we find that the DRM practice in Austria can be considered as early adaptation to climate change, addressing the existing adaptation deficit and mainstreaming climate change in decision processes (as, e.g., required by the European Union (EU) Flood Directive: DIRECTIVE 2007/60/EC; European Parliament and Council 2007). In turn, current and past public expenditures in the Austrian DRM field (BMLFUW and bmivt 2012) can be interpreted as expenditures for no-regret and low-regret early adaption measures and may serve as a first impression of how much money is being spent today to address the current adaptation deficit.

The key instrument for financing public disaster risk management in Austria is the Austrian disaster fund (BMF 2012). In addition to the annual endowment of the fund, which is dependent on tax revenues and hence on overall economic development, the fund can also draw on a built up reserve (BMF 2014). Originally accumulating in nature, the accumulation of reserves has been capped with the issuance of the current disaster fund law. Thus, in years where it was not necessary to withdraw funds from the reserve (as there were no major catastrophic events taking place in Austria or additional funds for ex post payments were available from extraordinary increases), surpluses from the disaster fund (deposits minus expenditures) were redistributed to the general budget as the build-up of the reserve was capped.

**Fig. 3:**
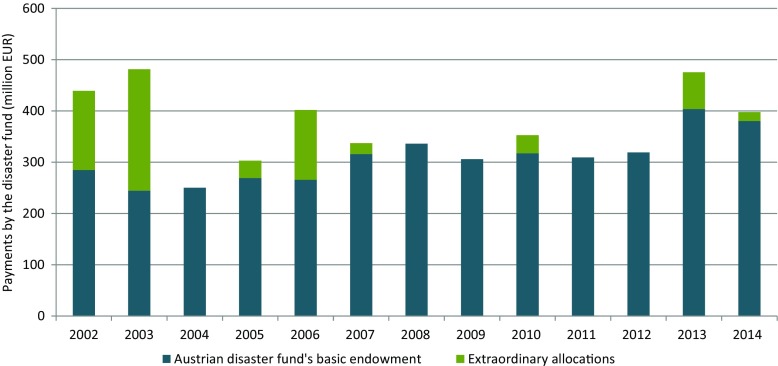
Payments by the Austrian disaster fund financed from its basic endowment, and from extraordinary allocations based on special laws (in 2002 and 2005) and federal government resolutions (since 2010), 2002-2014 (in million EUR). Data source: BMF (2014)

Severe floods in 2002, 2005, and 2013—with cost estimates for the 2002 and 2013 floods amounting to more than Euro (EUR) 3 billion (Habersack et al. 2004) and EUR 0.9 billion (BMI 2014), respectively—led to the situation that the fund’s usual resources (including the reserve) were not sufficient to cope with the losses of these catastrophic events. Hence, special-laws were enforced which provided an ad hoc increase of resources for the disaster fund. Figure 3 visualizes how these extraordinary allocations have been put to use.^2^ As a first reaction by the Austrian government, the federal funds provided via the disaster fund to the BMLFUW alone will be increased to annually 200 million EUR over the next five years (BMLFUW 2014).

### Climate risk modelling: Evaluating the fiscal burdens and required allocations to the disaster fund

**Fig. 4:**
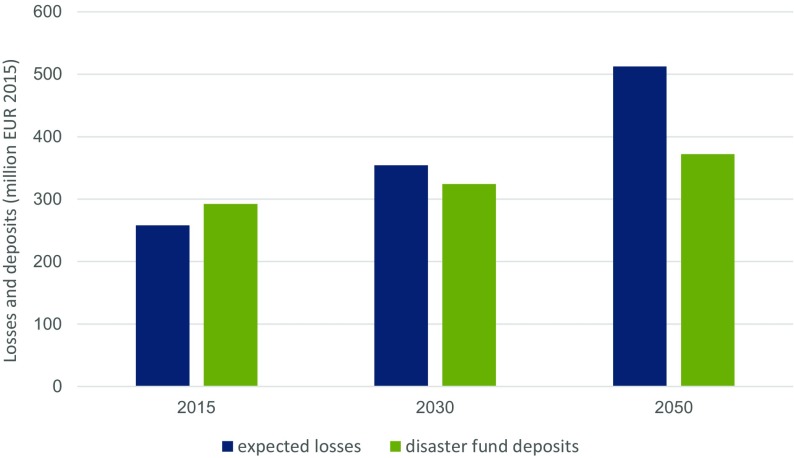
Development of expected annual losses from 2015 until 2030 and 2050 in contrast to the development of disaster fund deposits under business as usual (assuming a GDP growth rate of 1.7 % p.a. and an inflation rate of 1 % p.a) (in million EUR 2015)

Based on the empirical evidence, the next analytical step has been to project fiscal risk and compare it with budgetary allocations. In terms of risks to the Austrian disaster fund, we found that in 2015 the fund’s endowment, amounting to EUR 290 million, was still sufficient to cover the expected losses of EUR 260 million for this year (Fig. 4). According to actuarial science this would imply that the fund’s endowment is sufficient to cover the fair premium (i.e., the expected losses), which is a prerequisite to keep the fund positive in the long run. According to our modeling results the picture, however, changes until 2030, when contrasting the development of expected annual flood losses in Austria with the business as usual development of deposits in the Austrian disaster fund up to 2030 and 2050. Assuming a GDP growth rate of 1.7 % p.a. and an inflation rate of 1 % p.a. we estimated the endowment of the Austrian disaster fund amounting to EUR 320 million in 2030 and EUR 370 million in 2050. Neither in 2030 nor in 2050 this endowment will be sufficient to cover expected annual losses of EUR 350 million for 2030 and EUR 510 million for 2050, respectively, and severe stress could be put on the disaster fund’s financial resilience. Hence, Austrian decision-makers are well advised to understand that the public ex ante and ex post DRR expenditures will likely have to increase accordingly to given socio-economic and climate-related risk drivers to guarantee a sufficient level of protection from natural hazards and to remedy losses after a catastrophic event has occurred.

### Evaluation of the new normal according to risk layers

**Fig. 5:**
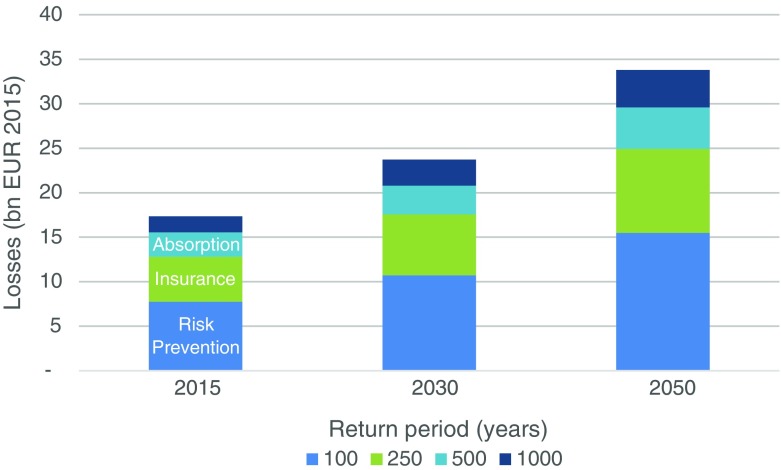
Probabilistic projections of flood losses (with flood protection measures) for different return periods in Austria based on a copula approach (in billion EUR 2015)

Results for different flood loss return periods (Fig. 5) also indicated an increase in losses up to 2050. Cumulated losses over the different return periods were estimated to increase from EUR 17 billion in 2015 to EUR 24 billion in 2030 and EUR 34 billion in 2050. It should be noted that the results are mainly driven by socioeconomic developments, leading to higher exposure of assets to flood risk, while climate change impacts are not found to be large in the near to medium future (the relative importance of climate change, exposure and vulnerability in driving risk is now a very active research area, see for example Mechler et al. (2014) for a discussion).

Probabilistic modeling results have been presented and discussed at a stakeholder workshop with the DRM and adaptation experts that had already been approached during the monitoring phase of our assessment, as well as with further participants active in this field. The main focus was on increasing participants understanding, of how a probabilistic modeling exercise may extend the analysis of risk by giving not only information about the changes in average losses but also about changes of the tails, i.e., extreme risk. Policy makers ought to also pay attention to the full loss distribution, particularly the tails of the distribution (high and very high return periods in Fig. 5). When talking about catastrophic events it is the low probability, high impact events that should matter most in decision making, as in case of occurrence such events could impose severe stress on federal budgets and can overburden risk instruments, such as the Austrian disaster fund, exactly at the moment when they are needed the most.

The fact that the losses caused by the floods in 2002, 2005, and 2013 put Austria’s main vehicle to finance DRR—the disaster fund—under pressure (Fig. 3), can be seen as a first evidence that the current approach to financing DRR in Austria is insufficient to sustainably cope with catastrophic climate-related events such as those in 2002 and 2013. The diversions from the general federal budget, which were necessary to provide additional resources for the Austrian disaster fund, put additional stress on the Austrian federal budget. Our analysis thus leads to the conclusion that the current, static approach to financing CRM in Austria is not an appropriate approach to manage low-frequency, high-impact events such as extreme floods and other climate risks in a broader understanding of CRM.

### Building blocks for an update of the Austrian CRM strategy at different layers of risk

Based on the climate risk modeling results and insights from their evaluation according to different layers of climate risks, we proceed by suggesting an update of the Austrian CRM strategy. For example, to tackle the 100-year return period layer of risk, we suggest to foster investments into preventive measures against flood risk in Austria. While the Austrian disaster fund has been an effective and well-functioning tool for risk reduction at this lower layer of risk, we still suggest some improvements to the current set-up of the fund. First, building-back-better damaged assets, which would serve the purpose of increasing resilience against future natural hazards, is not foreseen under the current form of Austria’s disaster fund. Providing financial assistance beyond the asset’s pre-catastrophe present value for rebuilding assets in a more catastrophe-proof fashion, would increase the effectiveness of preventive measures under the disaster fund as early adaptation measures dealing with existing and exacerbating climate variability and extremes. Second, while the preventive measures financed by the Austrian disaster fund today are almost exclusively physical measures, a more comprehensive approach to financing ex ante risk reduction should also incorporate tackling the underlying drivers of risk, e.g., by preventing the placement of capital (e.g., buildings) in flood-risk prone areas. The implementation of a national flood risk management plan, as required by the EU floods directive 2007/60/EC (European Parliament and Council 2007) is currently being established in Austria accompanied by a comprehensive stakeholder process. The draft version (BMLFUW 2015) proposes 22 measures along the full flood risk management cycle and has thus the potential to allow the broadening of the Austrian DRM praxis, moving beyond the pure focus on building measures as ex ante risk management and the remedying of losses as post-disaster relief measure, towards a more integrative approach to DRM. Third, in its current form, the Austrian disaster fund does not finance preventive measures in the private domain. Private households are only eligible to relief (up to a certain percentage of the asset’s pre-catastrophe present value) after a catastrophic event has occurred. Financing private preventive measures would change the incentive structure in the Austrian risk management practice further from a rather re-active to a pro-active approach and could prove highly cost effective in reducing climate-related risks.

For higher layers of risk, i.e., flood risks with, e.g., a 250-year return period, it is well worth considering to complement the disaster fund with natural catastrophe (NatCat) insurance systems to deal with private losses. Germany and Belgium are examples of countries that already have such NatCat insurance schemes in place, though with different nuances regarding the public-private character of the insurance system. Also for Austria, actionable models for NatCat schemes have been proposed, with the most comprehensive framework dating back to 2009 (Prettenthaler and Albrecher 2009). This model suggests a compulsory extension of the coverage of fire and or household insurances, as well as the pooling of different climate risks (flooding, landslide, earthquake, and avalanche). The establishment of a risk-bearing-community, comprising insured persons, insurers, re-insurers and a to-be-established NatCat Pool is suggested. Up to a system boundary, reflecting the annual loss-related requirements and the insurance ceiling, the NatCat risk-bearing-community scheme compensates for private losses. Beyond that ceiling a traditional disaster fund compensates further private losses. However, only private parties insured by the NatCat system should be eligible for a compensation via the disaster fund. Insurance premiums and deductibles should be diversified according to different flood risk zones.

Tackling even higher layers of climate risk, characterized by flood risk return periods of 500 years and beyond, we suggest to foster national and international risk financing and risk absorption schemes, such as the European Union Solidarity Fund (EUSF) or regional risk pools. The EUSF was established in 2002, a year of extensive flooding in Europe, as an ex post loss financing vehicle for EU member states and candidate countries (Aakre et al. 2010; Hochrainer et al. 2010; Hochrainer-Stigler et al. 2015). The EUSF, based on the latest reforms in 2014, pursues three major aims: to promote solidarity with those European countries having the least coping capacities regarding major disaster shocks; to contribute to proactive disaster risk reduction and DRM; and to foster its robustness regarding its own risk to depletion (Hochrainer-Stigler et al. 2015) In that way, low middle and high layers of risk can be tackled with appropriate instruments based on different needs perceived to be relevant from different stakeholders. Furthermore, by embedding risk layering within an iterative process as described in the following section, it is possible to evaluate and design today’s adaptation strategies (e.g., to close the adaptation gap) as well as to update these strategies for the future with evidence.

## Discussion and conclusions: a need for iteration—developing a generic iterative climate risk management strategy and methodological approach

Our study suggested a multi-method approach for the operationalization of CRM, and tested it using Austria as a case study. Here, we present and discuss the key specific and general lessons learnt for a more general application in research and practice.

### Informing reflexive and participatory debate

**Fig 6:**
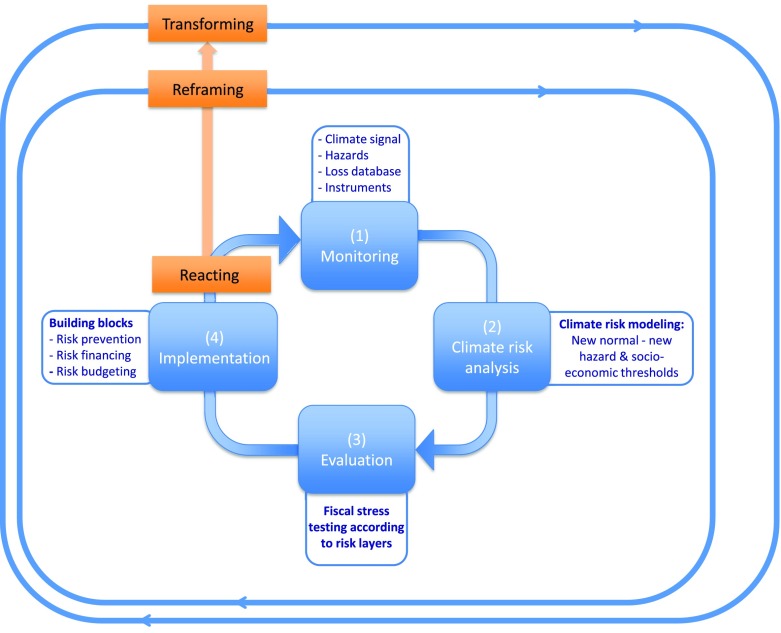
Iterative framework for climate risk management embedded in a triple loop learning process. Source: own figure, learning loop process adapted from Lavell et al. (2012)

Overall, we find that the CRM approach, co-developed with key national-level decision-makers, can serve as a useful reflexive-participatory framework to address the existing adaptation deficit and the uncertainties associated with future climate change impacts and losses in policy and practice. Moreover, as new knowledge on the complex dynamics of social–ecological systems and their interactions with a changing climate becomes available, this CRM framework can inform the required iterative update of current learning and CRM practice within a learning loop framework (Kolb and Fry 1975; Argyris and Schön 1978; Keen et al. 2005). Building on the results presented in the previous section, and based on the recent developments within the nexus of DRM and CCA research, the following generic operationalizable CRM framework (Fig. 6), which is applicable to other national and international decision-contexts, can be distilled.

At its core, our CRM framework (Fig. 6) consists of four steps and is embedded in a comprehensive participatory process, which at every stage requires thorough stakeholder involvement (e.g., DRM practitioners, the research community, affected communities, and representatives of ministries of finance, ministries of the interior and environmental ministries). Step (1) of our framework includes monitoring existing instruments, new scientific knowledge on climate change (e.g., emerging early trends and changes in variability that exacerbate existing risks or create new risks), natural hazard data (e.g., hydrological data), loss databases, and the climate signal. This is the basis for step (2): a model-based analysis of climate risks acknowledging the uncertainties associated with climate change in order to identify the new normal, which is characterized by new hazard-based and socioeconomic thresholds. This is followed by step (3): testing and evaluating the new normal according to different layers of climate risk, and potentially by an update of the measures already in place or the implementation of new instruments framed around the building blocks risk prevention, risk financing, and risk budgeting: step (4).

Similar to other national CRM practices, the Austrian CRM approach is currently characterized by a single-loop learning process, which is reacting to the new normal and focusing on improving the efficiency of current practices without questioning underlying mental models (Fig. 6). Based on our Austrian insights regarding flood risk, we argue that a new normal of climate risks may require a more fundamental reconsideration of the current approach, as well as a reframing of the problem and management goals within a double-loop learning process; the reason for this being that the current regimes in place might not be sustainable and resilient to anticipated shifts in flood losses. Moreover, in the longer term, the proposed CRM framework could even be extended by integrating entirely new approaches and additional parties into governance and participatory risk management in order to facilitate triple-loop learning. This approach to learning raises deep questions regarding the underlying principles of CRM, which could lead to a fundamental transformation of existing CRM practices, where planning is aimed at robust strategies rather than optimality (Pahl-Wostl 2009). In other words, this iterative approach to CRM allows for an adjustment of decisions over time with evidence and will eventually contribute to more robust policy response pathways to deal with the adaptation deficit and the future impacts of climate change.

Irrespective of the potential contribution of climate change to recent or future natural disaster losses and losses from extreme weather events, the most recent extreme event losses in Austria and elsewhere have revealed the urgent need for early adaptation, i.e., the need to deal with the current adaptation deficit based on current climate variability and weather extremes (see UNISDR 2015 for an assessment on the global scale). As part of an iterative CRM strategy, the international DRM practice will have to deal with these current and future climate risks in whatever way possible. In the medium to longer term, as new significant scientific evidence becomes available—in particular, research focusing on attributing natural hazards and associated losses to climate change—, climate change considerations might be incorporated in an iterative manner in the development of robust national and international adaptation pathways. This will require not only reconsidering how best to deal with losses from catastrophic events ex post but looking at future investments into preventive and protective ex ante measures—the eventual aim of ex ante DRR measures being to reduce ex post public liabilities.

### Informing instrumental debate

Many countries have a disaster fund or comparable disaster risk financing instruments in place. The fact that the losses incurred as a result of recent floods put Austria’s disaster fund under pressure, and required diversions from the general federal budget, can be seen as primary evidence that a static disaster fund approach to financing DRR may not be sufficient for coping with catastrophic climate-related events in a sustainable way. In fact, national governments are, implicitly (through moral obligations) and explicitly (through entitlements), taking over more and more climate risks on behalf of society. In combination with potentially reduced financial coping capacity under future socioeconomic and climate change developments, a continuation of this re-active approach to financing DRR would likely result in a reduction of governments’ fiscal spaces and may eventually lead to high opportunity costs as other socially desirable investments have to be forgone. One solution for counteracting these potential fiscal risks could be to link the development of deposits of a national disaster fund to ex ante estimates of future expected annual losses, and by doing so maintain a positive balance in the long run. However, as, e.g., the example of Mexico shows, this would not increase a country’s resilience to major natural disasters where losses significantly exceed AAL (Cardenas et al. 2007). Another strategy might be to set up a national disaster fund’s reserve policy as an accumulating, un-capped reserve. However, there are also caveats linked to any uncapped reserve, mainly arising in the form of opportunity costs associated with those funds set aside. Instead of having these reserves sitting idly^3^ in anticipation of, what are by definition, low probability catastrophic events, they could instead be employed to finance other socially and environmentally desirable investments. Eventually, these foregone investments may even contribute indirectly to a reduction in climate-related risks.

What is more, instead of relying on a single risk management tool, we suggest employing a more comprehensive and integrative approach to CRM. As there are different kinds of climate-related risks, some occurring frequently with only minor impacts while others occuring fairly infrequently but with devastating consequences (low and high return period events, respectively (see Fig. 5)), countries should employ a varied portfolio of instruments, each carefully chosen to be applicable for a certain layer of climate-related risk (Mechler et al. 2014) and, based on the evidence available, iteratively adjusted over time. For low layers of climate risk—characterized by high probability of occurrence but comparably low impacts—, risk reduction is often the most effective and cost efficient way forward. Ex ante preventive measures, such as constructing flood barriers, could be financed, e.g., through a disaster fund as in Austria. Medium layers of risk, with a lower probability of occurrence but in the event of an emergency lead to significantly more damaging (economic) impacts, may require alternative risk financing and risk transfer mechanisms if risk reduction possibilities are limited, e.g., natural catastrophe insurance. High layers of risk, with climate-related risks characterized by high return periods, mean that these risks are rare but catastrophic, requiring public post disaster assistance instead in order to absorb the manifested risks—as is the case in Austria, where it is financed by the Austrian disaster fund. However, given the potential for severe fiscal stress imposed by climate-related risks in the future, new and additional financing mechanisms may be needed. Internationally coordinated aid schemes, such as the EUSF, or international risk pools, are potentially highly cost-efficient and effective risk management instruments to deal with these high layers of risk.

### Challenges and opportunities

We based our analysis on multiple lines of evidence and methods—expert interviews and stakeholder workshops, empirical budget analyses, and risk-based modeling of past, current, and medium-term economic impacts of flood risks. While this comprehensive methodological toolbox is well-equipped to provide a complete picture of the current state of national CRM practices, future developments of climate risks and associated fiscal risks it is, at the same time, highly resource-intensive. This is particularly true in terms of budget data and the understanding and engagement with country-specific political and legal processes; the proposed approach requires a high level of commitment from all stakeholders involved. Nevertheless, our experience in Austria shows that this comprehensive, methods- and stakeholder-driven approach is highly relevant for designing robust national CRM pathways. With regard to the state of the art flood risk modeling approach employed in this study, it is important to note that the results are highly dependent on assumptions regarding flood protection levels. As for most flood models today, our flood loss calculations do not incorporate actual protection standards and, therefore, likely overestimate losses, especially for more frequent events. Ideally, one would use detailed information at the very local level to determine the specific protection level that actually exists at each location. However, this is not possible on larger scales (e.g., country level) as this kind of comprehensive information is not yet systematically available. To circumvent this problem, we used protection levels estimated in Jongman et al. (2014) which define flood protection standards as the minimum statistical probability of discharge that leads to flooding. Further research in this field is necessary in order to be able to generate more robust flood risk estimates. Applying the proposed iterative CRM framework, methods, and tools to other national contexts would be another natural fruitful area of future research. In doing so, this actionable approach to CRM itself can be iteratively adjusted and improved over time to eventually establish robust risk management pathways on a global scale.

Our methodological framework, case study findings and suggested building blocks for the practical implementation of an iterative CRM framework are of relevance beyond the case of Austria. Many countries and communities are feeling the impact of changes due to extreme events and are looking for robust strategies to reduce and manage the risks. Regions are developing improved approaches for absorbing increasing burdens, e.g., through the reformation of the European Solidarity Fund, or the establishment of regional risk pools for buffering against the financial risks from extremes in the Caribbean or Africa. Finally, the international community is committed to jointly tackling disaster risk based on the principle of moral responsibility via the Sendai mandate, as well as through the Warsaw International Mechanism on Loss & Damage (UNFCCC 2013). Fundamental to all these approaches is a broad-based and actionable perspective on CRM, which, we believe, will gain more traction in the years to come.

## Figures and Tables

**Fig. 7:**
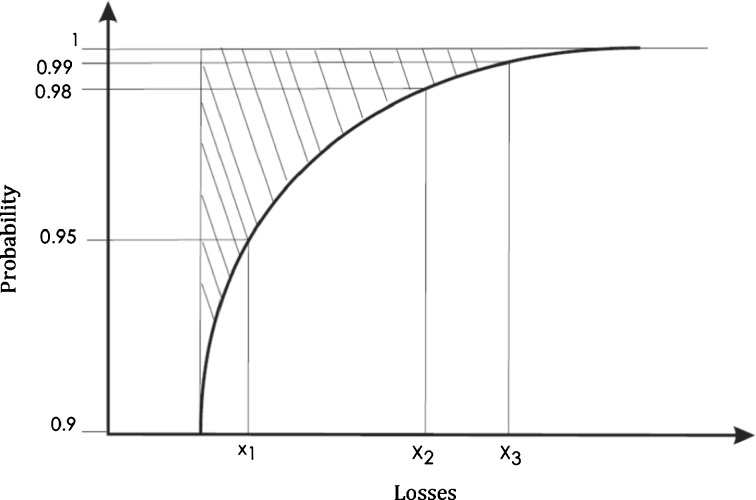
Generic loss distribution function: Cumulative distribution function with the *x*-axis representing losses (e.g., monetary losses, annual losses in terms of GDP, or capital stock losses), and the *y*-axis representing the probability that losses do not exceed a given level of damage

## Appendix

### Flood hazard modelling

Two methodological approaches are commonly used to estimate future losses due to climate and weather extremes: either a catastrophe modeling approach building on detailed analysis of physical hazards (Grossi and Kunreuther 2005), or the application of extreme value theory relying on past loss events (McNeil et al. 2005). Several flood hazard models at very local scale exist in Austria, however, currently only two flood risk modeling approaches provide country-level flood loss distributions. Full details of these models and discussion can be found in Prettenthaler et al. (2015). What is important for our discussion here is that in the first model (see Lugeri et al. 2010; Hochrainer-Stigler et al. 2014) the hazard module is not based on a dynamic hydrological model while the second one (Prettenthaler et al. 2015) performed the analysis via a bottom up-approach which did not include all exposure assets. We avoid both these limitations by using simulated losses based on the LISFLOOD hydrological model and an economic damage model (van der Knijff et al. 2010; Rojas et al. 2012). The flood loss data set has been validated in previous pan-European studies (Jongman et al. 2014) and provides therefore an ideal entry point for our analysis as we have been able to include climate change effects within one coherent approach. Note, that flood risk estimates (here in the form of loss distributions) from catastrophe modeling approaches are usually only available at GRID or basin scale (as in our case) and there is the additional challenge to up-scale these loss distributions to higher scales. Not until recently, information at larger scales on flood risk was only available for specific events or expressed in terms of average losses. Consequently, the full probabilistic risk information was not available anymore at these scales and risk management strategies could not be applied any longer (additionally averages do not give any information about the severity from extremes, see for a discussion Jongman et al. 2014).

In this paper we employ a risk based modeling tool, the IIASA CATSIM framework, to estimate future direct economic losses of extreme weather events and their repercussions on the Austrian federal budget. CATSIM follows the common practice in catastrophe models and evaluates the direct damages via three components or modules, i.e., the hazard, the exposure, and the vulnerability modules (Grossi and Kunreuther 2005). A fourth loss module summarizes the results from these modules with the help of risk metrics or loss distributions, which inform about the probability that losses do not exceed a given level of damage. Loss distributions are cumulative distribution functions where the x-axis represents the losses, e.g., monetary losses, annual losses in terms of GDP, or capital stock losses. The y-axis represents the probability that losses do not exceed a given level of damage. It therefore can be called the event axis. For example, in Fig. 7, a value of 0.98 on the event axis means that with a probability of 98 % the losses do not exceed a given level of damage, say *x*_2_. In other words, with a probability of 2 % the losses will exceed this level of damage. Note that a 2 % probability can be interpreted as a (1/0.02=) 50-year event, e.g., an event that happens on average once every 50 years. The same principle can be used for all other events. The loss distribution function itself is very useful for risk management purposes because various risk measures can be calculated from it (see Pflug and Römisch 2007). For example, the average annual loss, which is the area above the loss distribution, the Value at Risk (VaR) which is defined as VaR(*p*) = *F* ^−1^(1 − *p*), where *F* ^−1^ is the quantile function defined as the inverse of the loss distribution function, or the probable maximum loss (PML) which is associated with a given probability of exceedance (see also Grossi and Kunreuther 2005).

We perform a copula-based approach to derive probabilistic flood risk estimates at the country level for Austria. In more detail, we apply a structured coupling of probability loss distributions at the basin scale (derived from LISFLOOD; see van der Knijff et al. 2010; Rojas et al. 2012) based on a method discussed in Jongman et al. (2014) and more recently in Timonina et al. (2015). Dependencies between river basins in Austria are estimated using different copulas (e.g., Clayton, Frank, or Gumbel) and are based on maximum river discharges for the period 1990-2011. Afterwards, the loss distributions from each basin are coupled using the given copulas and a minimax ordering approach to finally derive a loss distribution at the country level. The details of the copula methodology, which is now seen as most appropriate to avoid underestimation of extreme risk (Jongman et al. 2014), and a general algorithm to perform such coupling can be found in Timonina et al. (2015). To the authors’ knowledge the only other model currently available for Austria employing a copula approach is the aforementioned one discussed in Prettenthaler et al. (2015), which, however, falls short in comprehensively including all exposed assets.

It is important to note here that, as in most flood models today, our flood loss estimates do not incorporate actual protection standards and therefore likely overestimate losses, especially for more frequent events. Ideally, one would use detailed information at the very local level to determine for each location the specific protection level which actually exists. However, this is not possible at higher scales (e.g., country level) as such comprehensive information is not systematically available yet. To circumvent this problem, we use protection levels estimated in Jongman et al. (2014) which defined flood protection standards as the minimum statistical probability of discharge that leads to flooding. The assessment was based on a large pan-European literature survey and modeling effort to establish estimates at the basin level for all European countries.

## Footnotes

1 We use disaster risk management (DRM) when referring to the operational aspects of disaster risk reduction (DRR) , i.e., the practical implementation of DRR measures.

2 While the funds from the increases based on the special laws in 2002 and 2005 had to be put to use within two and, respectively, three years, the increases based on the more flexible decision process as implemented by the 2008 fiscal equalization scheme had to be used in the very same year.

3 In the case of the Austrian disaster fund, the reserves have become a sole accounting identity since a reform in 2013 and no interest yields are accrued anymore since then from the invested reserve. Instead, the disaster fund’s reserves are regularly used for other public investment and expenditure items.
